# Is It Necessary to Remove the Maximum Prostate Tissue in All Patients? the Percentage of Resected Prostate Tissue and the Influence on Surgery Outcomes: A One‐Year Follow Up Study

**DOI:** 10.1002/nau.70152

**Published:** 2025-09-24

**Authors:** Bruno Rodrigues Lebani, André Barcelos da Silva, Luciano Teixeira Silva, Marcia Eli Girotti, Eduardo Remaile Pinto, Milton Skaff, Fernando Gonçalves Almeida

**Affiliations:** ^1^ Department of Surgery, Division of Urology, Voiding Dysfunction Section Federal University of São Paulo São Paulo Brazil

**Keywords:** bladder contractility index, bladder outlet obstruction, effectiveness, underactive bladder, urodynamics assessment

## Abstract

**Introduction:**

To investigate whether the volume of the prostate tissue resected on TURP influences on short and medium term follow up.

**Methods:**

It was developed a prospective study between May 2020 and August 2022, embracing patients with severe LUTS due to BPO, including clinical and urodynamic parameters meeting obstruction criteria (BOOI > 40), and good detrusor function (BCI > 100). Patients were assessed at 1, 6 and 12 months follow up. The primary endpoint was to compare whether the amount of resected tissue after TURP influences uroflowmetry at 12 months follow up (Qmax, ml/sec). The secondary endpoint was to compare different percentages of resected tissue (RPT) and its relation to the outcomes.

**Results:**

Ninety‐six patients with mean age of 70,4 ± 7.96 years. At baseline, prostate volume was 78.5 ± 51.8 cc³, Qmax was 6.03 ± 3.09 ml/sec and post void residual (PVR) was 113 ± 132 ml, IPSS of 24.9 ± 6.75. All of them were urodinamically obstructed (BOOI 86.7 ± 35.6) and good detrusor function (BCI 130 ± 28.6). The general RPT was 45.5 ± 27.7%. The higher the RTP, the lower the PSA at 1 month follow up (*p* < 0.001, R = 0.521). Nevertheless, it was not found correlation between the RTP and Qmax, IPSS or PVR.

**Conclusion:**

TURP improves clinical and urodynamic parameters at 1 year follow up, independent of the amount of resected prostate tissue, in patients with bladder outlet obstruction and good detrusor function, since the surgery is effective.

## 1 Introduction

Transurethral resection of the prostate (TURP) is still the gold‐standard surgical treatment for benign prostatic obstruction (BPO) in prostates smaller than 80 g [1]. Recently, there has been a shift towards maximal tissue resection to improve surgical outcomes and symptom relief. Enucleation of the adenoma using endoscopic techniques are associated with superior long‐term outcomes in terms of symptoms relief and obstruction resolution [2]. Nowadays, Holmium laser enucleation of the prostate (HoLEP) is the gold standard procedure to treat BPO, in any prostate size and mainly in glands larger than 80 g [1, 2]. However, these techniques often involve a steep learning curve and carry the risk of potentially severe complications, such as stress urinary incontinence, reported in up to 16% of patients 3 months postoperatively, even when performed by experienced surgeons [3].

The widespread use of conservative treatments for prostate enlargement, such as alpha‐blockers and 5‐alpha‐reductase inhibitors, has resulted in men undergoing surgical intervention at increasingly advanced ages. In such cases, life expectancy becomes a crucial factor to consider, like what is considered in prostate cancer guidelines. Different from uro‐oncological approaches, the current guidelines for benign prostatic hyperplasia (BPH) do not account for life expectancy when determining treatment strategies. This raises the question of whether surgical approaches, such as TURP, might be more suitable for patients with reduced life expectancy, particularly given TURP's widespread availability, lower stress urinary incontinence rates, and shorter learning curve. Is essential to remove all the adenoma to achieve good results? Are we considering more invasive and aggressive procedures on patients with reduced life expectancy?

There are few data about how much tissue must be resected during surgery to achieve good results. Moreover, data of the optimal volume of tissue resected during TURP are sparse and inconclusive. A previous published study suggested that removing less than 30% in TURP is sufficient to relieve urinary symptoms [4], as the criteria of unobstruction are met. However, the very short follow up (3 months) limits the reliability of its conclusions. On the other hand, another study suggested the greater the resected tissue weight of prostate, higher the improvement in symptoms scores, although confounding factors can interfere in results [5]. Furthermore, in those studies, there is no information regarding on level of obstruction or detrusor strength. In other words, probably the patients assessed on those studies represent a heterogenous population, including patients with underactive bladder. This lack of information can be understood assessing a more homogeneous population through urodynamic study.

The aim of this study was to assess whether the amount of prostate tissue resected influences on short and medium‐term follow‐up outcomes in patients with obstruction, assessed by pressure‐flow study.

## 2 Methods

### 2.1 Study Design

A prospective study, compromising 144 patients assessed for eligibility, was developed in a single center between May 2020 and August 2022, including subjects with severe lower urinary tract symptoms (LUTS) due to BPO refractory to conservative treatment. This study was performed according to the Declaration of Helsinki and approved by the ethics committee of Paulista School of Medicine—Federal University of São Paulo (CAAE: 37969020.6.0000.5505) [6]. All participants signed an informed consent form.

All patients underwent a detailed examination based on standardized clinical, radiological, laboratory, and urodynamic assessments. Baseline evaluations included clinical questionnaires, hemoglobin (Hb) levels, prostate‐specific antigen (PSA) tests, and post‐void residual volume (PVR). Lower urinary tract symptoms (LUTS) were assessed using the International Prostate Symptom Score (IPSS) questionnaire [7]. Prostate volume was measured via abdominal ultrasound and calculated as length × width × height × 0.52.

The inclusion criteria were patients with symptoms refractory to medical treatment, International Prostate Symptoms Score (IPSS) > 7, IPSS‐related quality of life (IPSSQoL) > 3, Bladder Outlet Obstruction index (BOOI) > 40, and good detrusor function with bladder contractility index (BCI) > 100. Urodynamic study was done in accordance with the International Continence Society good urodynamic practice [8]. Exclusion criteria included urethral stenosis, neurological conditions, prior prostate surgery prostate cancer. TURP were performed in all patients of the study under general or spinal anesthesia with a 26‐French Storz continuous‐flow resectoscope and a standard loop. The resected prostate tissue was weighed immediately after the surgical procedure without any liquid. Postoperatively, patients were assessed at 1‐, 6‐, and 12‐months follow‐up using

IPSS questionnaires, prostate‐specific antigen test (PSA), hemoglobin (Hb) and creatinine, post‐void residual measured by ultrasound (PVR).

The primary endpoint was to compare whether the amount of resected tissue after TURP influences uroflowmetry at 12 months follow‐up (Qmax ml/second). The secondary endpoint was to compare different percentages of resected tissue (RPT—resected prostate tissue = resected prostate tissue/prostate volume*100) and its relationship with outcomes according to groups:

- Group 1 – RPT > 60%
- Group 2 – RPT 30–60%
- Group 3 – RPT < 30%

### 2.2 Statistical Analysis

Statistical analysis was performed with JAMOVI software version 1.6. Pearson or Spearman tests were used for numeric variables according to data distribution. After the groups were divided according to the percentage of RPT, preoperative and postoperative data were assessed through ANOVA or Kruskal‐Wallis tests. Homogeneity was analyzed by Levene's test and data distribution by the Shapiro‐Wilk test.

## 3 Results

Ninety‐six patients were studied, after the exclusion of 48 patients which do not are suitable according to the inclusion and exclusion criteria. The mean age was 70.4 ± 7.96 years (mean ± standard deviation). At baseline, prostate volume was 78.5 ± 51.8 cc³, Qmax was 6.03 ± 3.09 ml/s, and post‐void residual (PVR) was 113 ± 132 ml. Patients had bothersome symptoms according to IPSS 24.9 ± 6.75. All of them were urodynamically obstructed (BOOI 86.7 ± 35.6) and had good detrusor function (BCI 130 ± 28.6). Prostatic specific antigen (PSA) was 5.07 ± 5.04 ng/ml. The general RPT was 45.5 ± 27.7%, as shown in Table 1.

**Table 1:** Demographic Data (Median ± Standard Deviation ‐ SD).

Baseline (96 patients)	Mean ± SD
**Age (Years)**	70.4 ± 7.96
**IPSS**	24.9 ± 6.75
**Prostate size (cc** ^**3**^ **)**	78.5 ± 51.8
**Qmax (ml/s)**	6.03 ± 3.09
**PVR (ml)**	113 ± 132
**BOOI**	86.7 ± 35.6
**BCI**	130 ± 28.6
**PSA (ng/ml)**	5.07 ± 5.04
Ressected prostate tissue (amount of tissue/total prostate volume *100)	45.5 ± 27.7

Abbreviations: BCI, bladder contractility index; BOOI, bladder outlet obstruction index; PSA, prostate‐specific antigen; PVR, post void residue; Qmax, maximum urinary flow on uroflowmtry.

**Figure 1:**
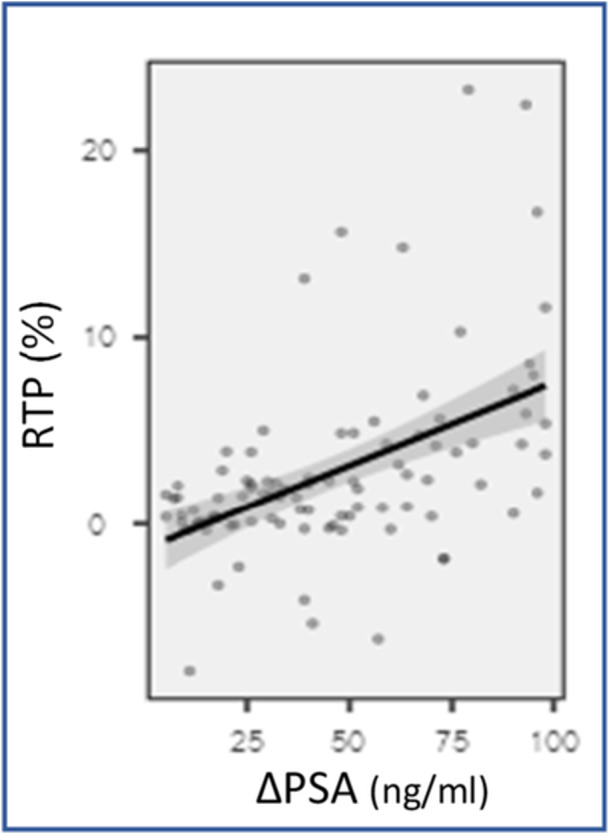
RTP vs ∆PSA (Spearman's test, *p* < 0.001 – *R* = 0.521; Linear regression with 95% confidence intervals.).

The higher the RPT, the lower the PSA at 1 month follow‐up (*p* < 0.001, *R* = 0.521), as shown in Figure 1. Furthermore, the higher the RTP, higher the hemoglobin variation. However, no correlation was found between the RPT and Qmax, IPSS, or PVR, as shown in Table 2, at 1, 6 and 12 months follow up.

**Table 2:** RTP and correlation with variables at 1 month follow up.

Data	p	R
**∆Qmax, ml/s**	0.802	0.026
**∆IPSS**	0.914	−0.011
**∆PSA, ng/ml**	< 0.001*	0.521
**∆PVR, ml**	0.769	0.035
**∆Hb, g/dl**	0.035*	0,220

Spearman's test;

* *p* < 0.001

∆Qmax Qmax variation baseline vs 1 month; ∆IPSS IPSS variation baseline vs 1 month; ∆PSA PSA variation baseline vs 1 month; ∆PVR PVR variation baseline vs 1 month; ∆Hb Hemoglobin variation baseline vs post‐operative.

**Figure 2:**
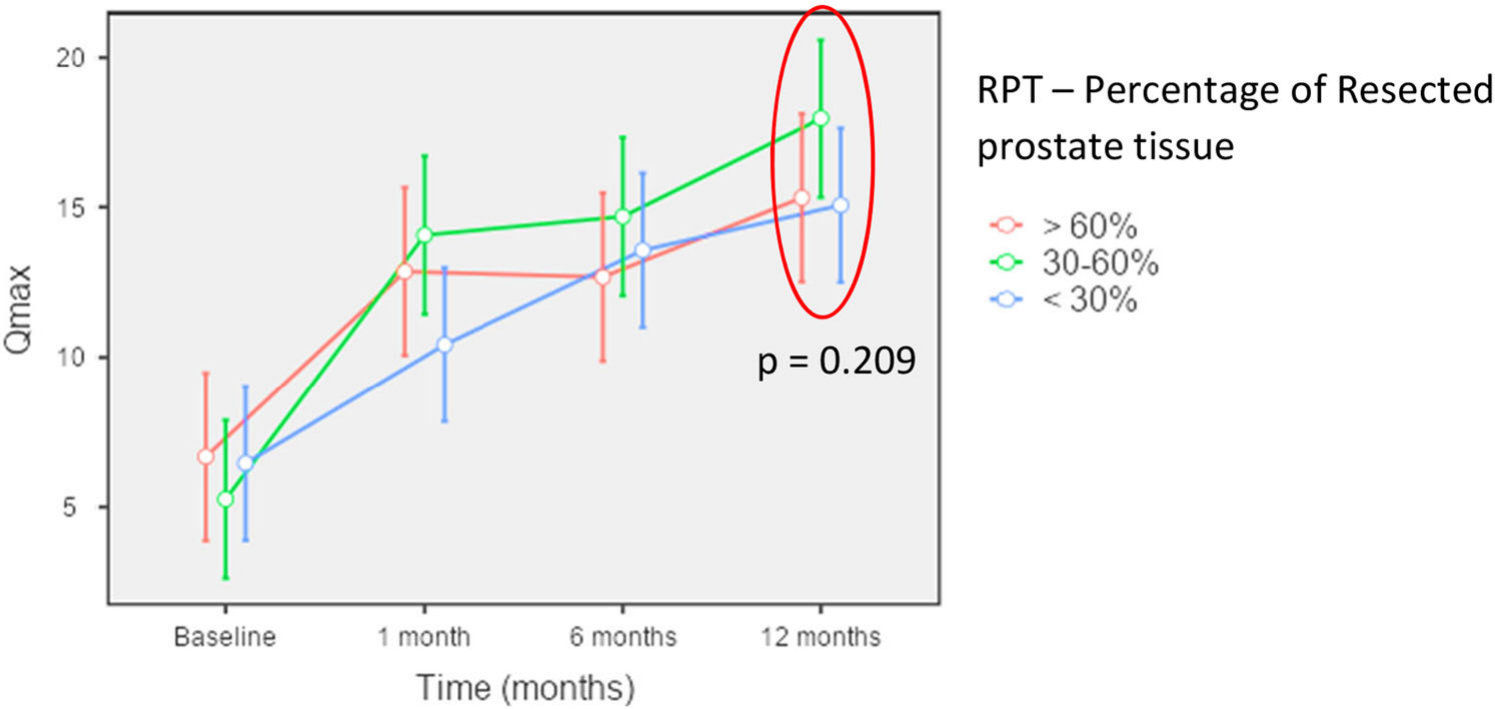
Qmax Variation (p refers to comparison among three groups at 12‐months follow up).

In sub analysis, according to the study design, three groups had a great improvement in Qmax compared with baseline, nevertheless with no difference among them (Kruskal‐wallis, *p* = 0209). It was seen similar results when analyzed IPSS and PVR, as shown in Table 3. Figure 2 shows Qmax variation in groups according to follow up.

**Table 3:** IPSS and PVR Preoperatively and Postoperatively Data (Median ± Standard Deviation; CI 95%.

Groups	Baseline		12 months		p (baseline vs 12 months)	
	IPSS	PVR	IPSS	PVR	IPSS	PVR
**1 – RTP > 60% (*n* = 28)**	25.1 ± 7.08	97 ± 90.9	5.41 ± 6.74	5.2 ± 12.9	< 0.001	< 0.001
**2 – RTP 30‐60% (*n* = 33)**	25.5 ± 7.14	159 ± 187	3.47 ± 3.52	4.92 ± 9.89	< 0.001	< 0.001
**3 – RTP < 30% (*n* = 34)**	24.1 ± 6.20	82.9 ± 78.6	3.97 ± 6.8	5.14 ± 13.3	< 0.001	< 0.001

There were no differences at 12 months follow up in IPSS, PVR (p respectively 0.388, 0.398 at 12 months follow up).

## 4 Discussion

There is a paucity of studies comparing the amount of prostatic tissue in TURP and achieved outcomes, with controversial results [4, 5]. Our study compared the amount of resected prostate tissue and its relation with subjective (measure by IPSS) and objective outcomes (measured by Qmax, PVR and PSA) in patients with bladder outlet obstruction and good detrusor function. At 12 months, we did not find association between the RTP and better outcomes, both objective and subjective.

These findings challenge the assumption that the primary goal of TURP is maximal tissue resection. We understand that a serious discussion is warranted with the patient, taking into account factors such as life expectancy and the potential risks associated with more aggressive surgical approach. The TURP aggressiveness can be verified with our study, assessing the hemoglobin drop (∆Hb), with statistical significance (the higher the RPT, the higher the Hb variation), although we must recognize the weakness of this conclusion (*R* = 0.22). Considering that BPO is a disease of the elderly, a more conservative surgery can be suitable for some patients, mitigating possible complications in the procedure.

Hakenberg et al. [5] revealed that the amount of resected tissue apparently affects the outcomes. Their study showed a negative correlation between the quantity of prostate resected and the postoperative symptom and bother scores at 3 months, suggesting that, there may be relation between these parameters. Nevertheless, a critical analyzes of their data revealed no clinically meaningful differences in outcomes during medium‐term follow‐up (6 and 12 months). Furthermore, it must be emphasized that their study did not perform urodynamics assessment before surgery, different from our findings. This discrepancy may explain the different patient profiles evaluated.

Conversely, Park et al. [9] observed different results. In their study, patients were stratified in 2 groups, according to the resection ratio (volume of resected tissue/prostate transitional zone −≥ 50% ou < 50%). They found no correlation between resected prostate tissue and improvement on IPSS, with both groups reaching similar outcomes. Antunes et al. [4] demonstrated that resection of less than 30% in TURP seems to be sufficient to alleviate urinary symptoms. Aagard and col found that patients who underwent partial resection of the prostate experienced similar improvements in voiding and storage symptoms, as well as in uroflowmetry and PVR [10]. We defined the RPT groups based on percentage tertiles of total prostate volume resected (<30%, 30–60%, >60%) and this stratification was chosen to allow comparisons across a wide volume range. Furthermore, none of these studies performed urodynamics assessments previously to invasive procedures, as we did.

There is literature supporting the detrusor strength and postoperative outcomes. Apparently, patients with underactive bladder had worst Qmax improvement than men with normal detrusor function [11, 12, 13]. This highlights and underscore the importance of urodynamics [14] in men undergoing surgery, essentially those with comorbidities or low life expectancy. Whether the detrusor function is adequate, a more conservative surgery could provide good improvements and could yield significant improvements in clinical and uroflowmetry outcomes, as well as in radiological parameters. It must be recognized that enucleation removes more tissue, however the goal of this study is to discuss whether such an approach is truly necessary for patients who may not benefit from long‐term results due to their age. Furthermore, endoscopic enucleation of the prostate still has adverse events which can deteriorate quality of life of patients, such as stress urinary incontinence [3, 15].

To our knowledge, this study represents the first comprehensive analysis of varying percentages of resected prostate tissue during transurethral resection of the prostate (TURP) specifically in patients who present bladder outlet obstruction and exhibit no signs of underactive detrusor function. We chose to stratify patients by the percentage of resected prostate tissue—rather than absolute gland size—to minimize the variability introduced by the wide range of prostate volumes in our cohort. This normalization strategy allowed us to assess resection efficacy individually, providing a more meaningful comparison across patients with diverse gland sizes. This data provides valuable insights that could inform surgical decision‐making and optimize treatment strategies for patients with BPO. It can be crucial, because it may lead to more tailored approaches that maximize patient benefits while minimizing potential risks, particularly in an aging population where the balance between surgical invasiveness and quality of life is vital.

There are some limitations in our study. Despite its prospective nature, it was not a randomized controlled trial, and the sample size is small. Therefore, the reliability of the conclusion becomes limited. However, the mentioned prospective design of the study enhances the robustness of the outcomes. We recognize that multivariable modeling can enhance the robustness of our findings by adjusting for confounding factors. However, we performed preliminary checks for key potential confounders (e.g., age, baseline prostate volume) and did not verify significant variability that would meaningfully interfere the interpretation of the results in univariable analyses. We acknowledge that future studies with larger cohorts may benefit from multivariable modeling to validate and expand on our findings.

Finally, we recognize a trend in current literature supporting prostate enucleation in patients with underactive detrusor (which was not the sample in this study) to reduce outlet resistance.

We understand that the life expectancy and other characteristics must be considered in the treatment of BPO, similar to protocols in prostate cancer that employ watchful waiting protocols. TURP eventually can be more conservative and quicker option, particularly in patients with severe comorbidities, or in cases which suboptimal results are acceptable, given that short‐term outcomes are comparable to those achieved through maximal resection. These data cannot be generalized to patients with underactive detrusor function.

## 5 Conclusion

TURP improves clinical and urodynamic parameters at 1 year follow up, independent of the amount of resected prostate tissue, in patients with bladder outlet obstruction and good detrusor function, since the surgery is effective, verified by satisfactory PSA drop. Life expectancy and comorbidities must be considered to perform a safe TURP, mitigating complications and adverse events.

## Author Contributions

All the authors have participated in the development of the study.

## Ethics Statement

This study was performed according to Declaration of Helsinque and approved byy the ethics committee of Paulista School of Medicine—Federal University of São Paulo (CAAE: 37969020.6.0000.5505). REBEC ‐ UTN U1111‐1250‐6616.

## Consent

The model applied to patients in this study is available and allows the permission to reproduce material from other sources.

This study is an assessment of our discipline database. The purpose of the study is to analyses the behavior of different percentages of resected prostate tissue during TURP and the outcomes. As it does not include new treatments, the clinical trial registration is unnecessary.

## Conflicts of Interest

The authors declare no conflict of interest.

## Permission to Reproduce Material From Other Sources

The authors allow to reproduce material from other sources.

## Data Availability

All the data are available to consultations whether and when required.
